# Supplementary material for: Redirecting the Immune Microenvironment in Acute Myeloid Leukemia
Source: Cancers (Basel). 2021 Mar 20;13(6):1423. doi: 10.3390/cancers13061423 (PMC8003817; doi:10.3390/cancers13061423)
Supplement: Supplementary file 1 [file cancers-13-01423-s001.pdf]

Supplementary Materials

Redirecting the Immune Microenvironment in Acute Myeloid Leukemia

Stephanie Sendker, Dirk Reinhardt and Naghmeh Niktoreh

Table S1. Checkpointinhibitor in AML. Selected clinical trials.

Immunotherapeutic-		Title Acronym Ref.	Phase	Status	Main Objectives	Population	Intervention Study Design	Outcome/ Response	TRAE
Target	Drug								
CTLA-4	Ipilimumab	NCT00060372 [1]	I	completed	Safety dose pharmacokinetics dosing regime Efficacy	Adults persistent/progressive cancer after allo-SCT (n = 29) incl. AML (n = 2)	Single arm: Ipilimumab + DLI	Objective response: CR (2/29) PR (1/29) No objective response for AML (n = 2) [1]	IAE (4/29), incl. AML (n = 1): grade 3 arthritis [1]
		NCT01822509 [2] [3]	I/Ib	Active, not recruiting	MTD Toxicity at MTD safety OS (1y), PFS	Adults relapsed hematologic malignancies after allo-SCT (n = 28) incl. AML (n = 12)	Single arm: Ipilimumab (3mg/kg, n = 6 or 10mg/kg, n = 22) or Nivolumab	3mg/kg: No formal response 10mg/kg: Complete Response (5/22) 23%) Partial response ((2/22) 9%) [2]	IAE (6/28, (21%), incl. 2 ⩾ grade 3 IAE and 1 death) DLT-GVHD (4/28 (14%)) [2]

						Adults relapsed hematologic malignancies after allo-SCT (n = 29)		ORR (32%)<br>PFS (23%)<br>OS (56%) [3]	DLT-IAE (2/6 1mg/kg), 2/22 (0,5mg/kg)<br>DLT-GVHD 2/22 (0,5mg/kg) [3]
		NCT02464657 [4]	I/II	completed	MTD, DLT<br>EFS	Adults<br>AML (n = 42) / High risk MDS (n = 2)	Single arm: Idarubicin +Cytarabine +Nivolumab	ORR (34/44 (77%)) (incl. 28 CR, 6 CRi) [4]	IAE ($\geqslant$grade 3)6/44 [4]
**PD-1**	Nivolumab	NCT02397720 [5]	II	recruiting	MTD<br>DLT<br>ORR	Adults<br>r/r or newly diagnosed AML (n = 53)	Non-Randomized: nivolumab +azacitidine versus nivolumab +ipilimumab +azacitidine	ORR (35%) (Incl. CR/CRi 11/53 (21%)) [5]	Immune toxocities ($\geqslant$grade 3) 7/53 (14%) [5]
		NCT03825367	I/II	recruiting	DLT<br>CR	Children/ young adults (1 -30 y.) r/r AML	Single arm: Nivolumab and 5-azacytidine	N/A	N/A

Table S2. Antibody-based Immunotherapies in AML. Selected clinical trials.

Immunotherapeutic-			Title Acronym Ref.	Phase	Status	Main Objectives	Population	Intervention study design	Outcome/Response	TRAE
**Principle**	**Target**	**Drug**								
**ADC**	CD33	Gemtuzumab -Ozogamicin	NCT00927498 [6]	III	completed	EFS<br>CR<br>OS<br>Safety	untreated adult AML (n = 280)	Randomized<br>Arm I: Daunorubicin and Cytarabine<br>Arm II: Daunorubicin and Cytarabine +GO	CR n = 217 (202 CR, 15 CRp) (104 Arm1, 113 Arm2) [6]	N/A
			NCT00372593 [7]	III	completed	EFS<br>OS (3y)	Pediatric and young patients (0 – 29 y.) newly diagnosed AML (n = 1022)	Randomized<br>Arm I: conventional combination chemotherapy versus Arm II: conventional combination chemotherapy +GO	EFS: 46.9% vs. 53.1%<br>OS (3y): 65.4% vs. 69.4%<br>RR: 41.3 vs. 32.8%<br>remission: 88% vs. 85% [7]	SAE (⩾ grade 3)<br>Arm A: 26/511 (5.09%)<br>Arm B: 32/511 (6.26%)
	CD123	IMGN362	NCT03386513 [8]	I/II	recruiting	MTD<br>RP2D<br>ORR	Adult r/r hematologic malignancies, incl. AML (n = 66)	Single agent, single arm: IMGN362	objective response 13 (20%) (3 CR, 8 CRi)<br>RP2D: 0.045 mg/kg (day 1, q3w) [8]	infusion-related reactions (16%; grade 3) [8]
**ADCC**		CSL362	NCT01632852 [9]	I	completed	AE<br>DLT	Adult CD123+AML in CR/CRp at High Risk	Single agent, single arm	3mg/kg durable saturation 10/20 maintained OR for 6 month [9]	3/25 DLT (incl. 2 pt. IR) [9]
		CSL362 JNJ-56022473	NCT02992860 [10]	II	Terminated [1]	ORR	Elderly pt. HR-MDS (n = 5), AML failing HMA (n = 19)	Single agent, single arm	ORR 2/25 (8%) (incl. 1 CRi, 22 weeks; 1 HI-E, 8 weeks) [10]	Significant toxicity (incl. death n = 6) [10]
**BiAB**	CD123 x CD3	Flotetuzumab DART® MGD006	NCT02152956 [11]	I/II	Recruiting	CR<br>MTD	Adult r/r AML (n = 88) (Dose finding n = 42; RP2D treated n = 50)	Single agent, single arm: floteluzumab	ORR 12/88 (13.6%)<br>CR 10/88 (11.7%)<br>MTD 500ng/kg/d [11]	IRR/CRS 7/88 (8%) [11]

[1] Enrollement was stopped due to a recommendation by the IDMC/FDA for a parallel clinical trial with TALA, where potentielly no efficancy could be determined.

Table S3. Cellular- and Cytokine-based Immunotherapies in AML. Selected clinical trials.

Immunotherapeutic-			Title Acronym Ref.	Phase	Status	Main Objectives	Population	Intervention Study Design	Outcome/ Response	TRAE
**Principle**	**Target**	**Drug**								
**CAR**	CD33	CD33CART	NCT03971799	I/II	Recruitting	MTD Morphological remission (d28)	Children and young adults r/r AML	Single agent, single arm: CD33CART	N/A	N/A
		Anti-CD33 CAR-NK cells	NCT02944162 [12]	I/II	unknown	Adverse events ORR	Adult r/r AML	Single agent, single arm: CD33CART	N/A	N/A
**Cytokine induced DLI**	NK-cell + T-cell	CIML NK Cell	NCT03068819 [13]	I/II	recruiting	Feasibility, Safety (i.e. unexp. GVHD) LFS	pediatric (1 – 18 y.)/ young adult and adult cohort r/r AML after allo-SCT	Non-Randomized: CIML NK Cell Infusion (d0) + CD3+ T Cell Product (d-1)	N/A	N/A
	NK-cell + IL-15 / + IL-2	CIML NK Cell Adoptive Therapy + ALT-803	NCT01898793	I/II	Recruiting	MTD CR/CRi Safety (AE)	Adult and pediatric (2 – 18 y.)/ young adult cohort r/r AML or HR-MDS	Non-Randomized: +Leukapheresis + cytokine induced NK cells + ALT-803 / IL-2 (Ph 1)	N/A	N/A

		NCT02782546	II	Recruiting	LFS	Adult r/r AML after haplo-SCT	Non-Randomized: allo-SCT + G-CSF CIML NK cell infusion + ALT-803	N/A	N/A
NK-cell + IL-15	Haplo NK-cell + SubQ rhIL-15	NCT02395822 [14]	II	completed	Efficacy to achieve CR	Adult r/r AML (*n* = 17, completed *n* = 15)	Single arm: IL-15 activated GMP-Donor NK Cells + s.c. rHIL-15	OR: 6/16 (40%) CR: 1/16 (6%) CRi:5/16 (33%) PD: 9/16 (60%)	CRS (grade ⩾ 3) 5/17 (30%) Serious AE 12/17 (71%)
	Haplo NK-cell + i.V. rhIL-15	NCT01385423 [14]	I	completed	MTD, DLT	Adult r/r MDS, AML (*n* = 26, completed *n* = 25)	Single arm: IL-15 activated GMP-Donor NK Cells + i.v. rHIL-15	OR: 8/26 (32%) CR: 6/26 (24%) CRi:2/26 (8%) PD: 17/26 (68%) [14]	CRS (grade ⩾ 3) 0/27 [14]

Table S4. Vaccine-based Immunotherapies in AML. Selected clinical trials.

Immunotherapeutic-.			Title Acronym Ref.	Phase	Status	Main Objectives	Population	Intervention Study Design	Outcome/ Response	TRAE
Principle	Target	Drug								
Peptide vaccine	WT-1	Galinpepimut-S	NCT01266083 [15]	II	completed	Safety (toxicity) Efficacy (3y.)	Adult ALL, AML in CR (*n* = 22)	Single arm: WT1 peptide vaccine	OS (3y) not reached, estimated ⩾67.6 [15]	Total toxicities events (⩾ grade 3) 94/244 (38,5%) [15]
			NCT04229979	III	recruiting	OS (up to 52 weeks)	Adult AML CR2/CRp2	Randomized: Galinpepimut-S vs. best available treatment	N/A	N/A
Dendritic cell vaccine		WT1 mRNA-electroporated Autologous Dendritic Cell Vaccination	NCT00965224 [16]	II	unknown	Immunogenicity Induction /maintenance of molecular remission (MR)	Adult CML, multiple myeloma, AML in remission at High risk (*n* = 30)	standard therapy + vaccination vs. no intervention (standard therapy)	MR (normalized WT1 mRNA level) *n* = 9 >1.5-fold increased WT1-specific CD8$^+$ T cell response 6/12 (evaluable HLA-A*0201$^+$) [16]	N/A
	hTERT	GRNVAC1 (hTERT-DC)	NCT00510133 [17]	II	completed	Feasibility (1y)	Adult AML in cCR	Single arm: GRNVAC1 Autologous dendritic cell vaccine	Eligible for hTERT - DC vaccination 21/33 (64%) [17]	SAE (⩾ grade3) 6/21 (29%) [17]

Table S5. Oncolytic Viruses in AML. Selected clinical trials.

Immunotherapeutic-		Title Acronym	Phase	Status	Main Objectives	Population	Intervention Study Design	Outcome/ Response	TRAE
Principle	Drug								
**Oncolytic-VSV**	VSV-hIFNbeta-NIS	NCT03017820	I	recruiting	MTD, AE (⩾grade 3)	Adult r/r Multiple Myeloma, AML, T-cell Lymphoma	Arm A: VSV-hIFNbeta-NIS<br>Arm B: + ruxolitinib phosphate<br>(Arm C: + ruxolitinib phosphate + cyclophophamide in r/r multiple myeloma)	N/A	N/A

Abbreviations: Treatment related adverse events (TRAE). Immune related adverse events (IAE). Adverse events (AE). Not applicable (N/A). Relapsed/ refractory (r/r). Dose-limiting toxicity (DLT). Objective response rate (ORR). Progression-free survival (PFS). Maximum tolerated dose (MTD). Leukemia free survival (LFS). Recommended Phase 2 dose (RP2D). Overall survival (OS). Event free survivial (EFS). Complete response (CR). Complete response with incomplete blood recovery (CRi). Clinical complete response (cCR). Progressive disease (PD). Gemtuzumab-Ozogamicin (GO). Antibody-drug conjugate (ADC). Antibody-dependent cellular cytotoxicity (ADCC). Bispecific Antibody (BiAb). Donor lymphocyte infusion (DLI). Cytokine release syndrome (CRS). Cytokine-induced memory-like (CIML). Human telomerase reverse transcriptase (hTERT). Molecular remission (MR). Dendritic cell (DC). Vesicular stomatitis virus (VSV).

References

1. Bashey, A.; Medina, B.; Corringham, S.; Pasek, M.; Carrier, E.; Vrooman, L.; Lowy, I.; Solomon, S.R.; Morris, L.E.; Holland, H.K.; et al. CTLA4 blockade with ipilimumab to treat relapse of malignancy after allogeneic hematopoietic cell transplantation. *Blood* **2009**, *113*, 1581–1588, doi:10.1182/blood-2008-07-168468.
2. Davids, M.S.; Kim, H.T.; Bachireddy, P.; Costello, C.; Liguori, R.; Savell, A.; Lukez, A.P.; Avigan, D.; Chen, Y.-B.; McSweeney, P.; et al. Ipilimumab for Patients with Relapse after Allogeneic Transplantation. *N. Engl. J. Med.* **2016**, *375*, 143–153, doi:10.1056/NEJMoa1601202.
3. Davids, M.S.; Kim, H.T.; Costello, C.; Herrera, A.F.; Locke, F.L.; Maegawa, R.O.; Savell, A.; Mazzeo, M.; Anderson, A.; Boardman, A.P.; et al. A multicenter phase 1 study of nivolumab for relapsed hematologic malignancies after allogeneic transplantation. *Blood* **2020**, *135*, 2182–2191, doi:10.1182/blood.2019004710.
4. Assi, R.; Kantarjian, H.M.; Daver, N.G.; Garcia-Manero, G.; Benton, C.B.; Thompson, P.A.; Borthakur, G.; Kadia, T.M.; Alvarado, Y.; Jabbour, E.J.; et al. Results of a Phase 2, Open-Label Study of Idarubicin (I), Cytarabine (A) and Nivolumab (Nivo) in Patients with Newly Diagnosed Acute Myeloid Leukemia (AML) and High-Risk Myelodysplastic Syndrome (MDS). *Blood* **2018**, *132*, 905, doi:10.1182/blood-2018-99-116078.
5. Daver, N.; Basu, S.; Garcia-Manero, G.; Cortes, J.E.; Ravandi, F.; Jabbour, E.J.; Hendrickson, S.; Pierce, S.; Ning, J.; Konopleva, M.; et al. Phase IB/II Study of Nivolumab in Combination with Azacytidine (AZA) in Patients (pts) with Relapsed Acute Myeloid Leukemia (AML). *Blood* **2016**, *128*, 763, doi:10.1182/blood.V128.22.763.763.

6. Renneville, A.; Abdelali, R.B.; Chevret, S.; Nibourel, O.; Cheok, M.; Pautas, C.; Duléry, R.; Boyer, T.; Cayuela, J.-M.; Hayette, S.; et al. Clinical impact of gene mutations and lesions detected by SNP-array karyotyping in acute myeloid leukemia patients in the context of gemtuzumab ozogamicin treatment: Results of the ALFA-0701 trial. *Oncotarget* **2014**, *5*, 916–932, doi:10.18632/oncotarget.1536.
7. Gamis, A.S.; Alonzo, T.A.; Meshinchi, S.; Sung, L.; Gerbing, R.B.; Raimondi, S.C.; Hirsch, B.A.; Kahwash, S.B.; Heerema-McKenney, A.; Winter, L.; et al. Gemtuzumab ozogamicin in children and adolescents with de novo acute myeloid leukemia improves event-free survival by reducing relapse risk: Results from the randomized phase III Children's Oncology Group trial AAML0531. *J. Clin. Oncol.* **2014**, *32*, 3021–3032, doi:10.1200/JCO.2014.55.3628.
8. Daver, N.G.; Montesinos, P.; DeAngelo, D.J.; Wang, E.S.; Papadantonakis, N.; Deconinck, E.; Erba, H.P.; Pemmaraju, N.; Lane, A.A.; Rizzieri, D.A.; et al. Clinical Profile of IMGN632, a Novel CD123-Targeting Antibody-Drug Conjugate (ADC), in Patients with Relapsed/Refractory (R/R) Acute Myeloid Leukemia (AML) or Blastic Plasmacytoid Dendritic Cell Neoplasm (BPDCN). *Blood* **2019**, *134*, 734, doi:10.1182/blood-2019-128648.
9. Smith, B.D.; Roboz, G.J.; Walter, R.B.; Altman, J.K.; Ferguson, A.; Curcio, T.J.; Orlowski, K.F.; Garrett, L.; Busfield, S.J.; Barnden, M.; et al. First-in Man, Phase 1 Study of CSL362 (Anti-IL3Rα / Anti-CD123 Monoclonal Antibody) in Patients with CD123+ Acute Myeloid Leukemia (AML) in CR at High Risk for Early Relapse. *Blood* **2014**, *124*, 120, doi:10.1182/blood.V124.21.120.120.
10. Kubasch, A.S.; Schulze, F.; Götze, K.S.; Krönke, J.; Sockel, K.; Middeke, J.M.; Chermat, F.; Gloaguen, S.; Puttrich, M.; Weigt, C.; et al. Anti-CD123 Targeted Therapy with Talacotuzumab in Advanced MDS and AML after Failing Hypomethylating Agents—Final Results of the Samba Trial. *Blood* **2018**, *132*, 4045, doi:10.1182/blood-2018-99-113112.
11. Uy, G.L.; Godwin, J.; Rettig, M.P.; Vey, N.; Foster, M.; Arellano, M.L.; Rizzieri, D.A.; Topp, M.S.; Huls, G.; Lowenberg, B. Preliminary results of a phase 1 study of flotetuzumab, a cd123 x cd3 bispecific dart® protein, in patients with relapsed/refractory acute myeloid leukemia and myelodysplastic syndrome. *Blood* **2017**, *130*, 637.
12. Tang, X.; Yang, L.; Li, Z.; Nalin, A.P.; Dai, H.; Xu, T.; Yin, J.; You, F.; Zhu, M.; Shen, W.; et al. First-in-man clinical trial of CAR NK-92 cells: Safety test of CD33-CAR NK-92 cells in patients with relapsed and refractory acute myeloid leukemia. *Am. J. Cancer Res.* **2018**, *8*, 1083–1089.
13. Bednarski, J.J.; Zimmerman, C.; Cashen, A.F.; Desai, S.; Foster, M.; Schappe, T.; McClain, E.; Becker-Hapak, M.; Berrien-Elliott, M.M.; Fehniger, T.A. Adoptively Transferred Donor-Derived Cytokine Induced Memory-like NK Cells Persist and Induce Remission in Pediatric Patient with Relapsed Acute Myeloid Leukemia after Hematopoietic Cell Transplantation. *Blood* **2019**, *134*, 3307, doi:10.1182/blood-2019-126982.
14. Cooley, S.; He, F.; Bachanova, V.; Vercellotti, G.M.; Defor, T.E.; Curtsinger, J.M.; Robertson, P.; Grzywacz, B.; Conlon, K.C.; Waldmann, T.A.; et al. First-in-human trial of rhIL-15 and haploidentical natural killer cell therapy for advanced acute myeloid leukemia. *Blood Adv.* **2019**, *3*, 1970–1980, doi:10.1182/bloodadvances.2018028332.
15. Maslak, P.G.; Dao, T.; Bernal, Y.; Chanel, S.M.; Zhang, R.; Frattini, M.; Rosenblat, T.; Jurcic, J.G.; Brentjens, R.J.; Arcila, M.E.; et al. Phase 2 trial of a multivalent WT1 peptide vaccine (galinpepimut-S) in acute myeloid leukemia. *Blood Adv.* **2018**, *2*, doi:10.1182/bloodadvances.2017014175.
16. Anguille, S.; van de Velde, A.L.; Smits, E.L.; van Tendeloo, V.F.; Juliusson, G.; Cools, N.; Nijs, G.; Stein, B.; Lion, E.; van Driessche, A.; et al. Dendritic cell vaccination as postremission treatment to prevent or delay relapse in acute myeloid leukemia. *Blood* **2017**, *130*, 1713–1721, doi:10.1182/blood-2017-04-780155.
17. Khoury, H.J.; Collins, R.H.; Blum, W.; Stiff, P.S.; Elias, L.; Lebkowski, J.S.; Reddy, A.; Nishimoto, K.P.; Sen, D.; Wirth, E.D.; et al. Immune responses and long-term disease recurrence status after telomerase-based dendritic cell immunotherapy in patients with acute myeloid leukemia. *Cancer* **2017**, *123*, 3061–3072, doi:10.1002/cncr.30696.
